# Ecometabolomic Analysis of Wild Populations of *Pilocarpus pennatifolius* (Rutaceae) Using Unimodal Analyses

**DOI:** 10.3389/fpls.2019.00258

**Published:** 2019-03-06

**Authors:** Daniella M. Allevato, Eduardo Kiyota, Paulo Mazzafera, Kevin C. Nixon

**Affiliations:** ^1^Section of Plant Biology, School of Integrative Plant Science, Cornell University, Ithaca, NY, United States; ^2^Departamento de Biologia Vegetal, Instituto de Biologia, Universidade Estadual de Campinas, Campinas, Brazil; ^3^Departamento de Produção Vegetal, Escola Superior de Agricultura Luiz de Queiroz, Universidade de São Paulo, Piracicaba, Brazil

**Keywords:** ecological metabolomics, unimodal ordination, *Pilocarpus pennatifolius*, chemoecotype, ecophysiology, canonical correlation analyses

## Abstract

Studies examining the diversity of plant specialized metabolites suggest that biotic and abiotic pressures greatly influence the qualitative and quantitative diversity found in a species. Large geographic distributions expose a species to a great variety of environmental pressures, thus providing an enormous opportunity for expression of environmental plasticity. *Pilocarpus*, a neotropical genus of Rutaceae, is rich in alkaloids, terpenoids, and coumarins, and is the only commercial source of the alkaloid pilocarpine for the treatment of glaucoma. Overharvesting of species in this genus for pilocarpine, has threatened natural populations of the species. The aim of this research was to understand how adaptation to environmental variation shapes the metabolome in multiple populations of the widespread species *Pilocarpus pennatifolius*. LCMS data from alkaloid and phenolic extracts of leaf tissue were analyzed with environmental predictors using unimodal unconstrained and constrained ordination methods for an untargeted metabolomics analysis. PLS-DA was used to further confirm the chemoecotypes of each site. The most important variables contributing to the alkaloid variation between the sites: mean temperature of wettest quarter, as well as the soil content of phosphorus, magnesium, and base saturation (V%). The most important contributing to the phenolic variation between the sites: mean temperature of the wettest quarter, temperature seasonality, calcium and soil electrical conductivity. This research will have broad implications in a variety of areas including biocontrol for pests, environmental and ecological plant physiology, and strategies for species conservation maximizing phytochemical diversity.

## Introduction

The vast array of compounds synthesized in plants has led to much debate over the function or adaptive significance for such a diversity of compounds. When plant specialized metabolites (PSM) were first discussed, they were often seen as waste products from primary metabolism, accidents or aberrant metabolism (Haslam, 1986). Today, PSMs are believed to have multiple functions and have been shown to be as important as primary metabolites in many cases (Wink, 1999; Cipollini, 2000; Izhaki, 2002).

The qualitative and quantitative PSM diversity in plants appears to change over time (phenology) and space, encompassing many ecological roles (Moore et al., 2014). Studies examining the diversity of PSM suggest that in the environment, biotic and abiotic pressures greatly influence the qualitative and quantitative diversity of the metabolome found in a species. The Climatic Variability hypothesis suggests that locations with constant warm temperatures and little seasonal variation would lead to a lower capacity for environmental plasticity (Molina-Montenegro and Naya, 2012). Large geographic distributions expose a species to a great variety of environmental pressures, thus providing an opportunity for an enormous range of chemoecotypes (Sultan, 1987; Tack et al., 2012; Gratani, 2014).

*Pilocarpus*, a genus of Rutaceae in the sub-tribe Pilocarpine, is known for its high bioactivity due to the presence of many alkaloids, terpenoids and coumarins (Santos and Moreno, 2004). Specifically, the genus has been sought after for one specific imidazole alkaloid, pilocarpine, as it is the only source of this compound that is used to treat glaucoma, as well as xerostomia. The genus *Pilocarpus* is the only known genus that contains this compound, and many of the species in this genus have become threatened with extinction due to overharvesting of wild populations (Pinheiro, 1997, 2002). Besides two chemical variants found in the genus *Casimoroa* (Rutaceae), Imidazole alkaloids have not been found in any other genus of plants. The genus *Pilocarpus* comprises 16 species and has a widespread, but strictly Neotropical distribution from southern Mexico and the Antilles, to northern Argentina (Skorupa, 1996).

Previous studies on other species of *Pilocarpus* have found that abiotic factors such as environmental variation can affect compound expression (Abreu et al., 2007; Sawaya et al., 2011). One study examining imidazole alkaloid concentrations in leaf tissue in *Pilocarpus microphyllus* plantations in Southern Brazil has found that there is variation of compound concentrations and compound presence depending on the season. The exposure to seasonality in the South is very different from the uniform seasons where *P. microphyllus* is naturally found, in the tropical rainforest and terra firme regions of the Amazonian state of Para. The seasonal variation of alkaloid production in *P. microphyllus* has demonstrated a strong association between specific groups of compounds and seasons, and has led to the hypothesis that distinct biosynthetic pathways are active in different seasons (Abreu et al., 2007; Sawaya et al., 2011). In addition, studies looking at salt stress, wounding, and hypoxia in *Pilocarpus* reported significant reductions in certain compounds following exposure to these conditions, further supporting the plasticity of certain genotypes to environmental stresses (Avancini et al., 2003). Further studies using *P. microphyllus* calluses and seedlings showed variation in pilocarpine production with pH variation, absence/excess of nutrients N, K, P, and NaCl, and amino acid precursors histidine and threonine (Avancini et al., 2003; Abreu et al., 2005; Andreazza et al., 2009).

One species in the genus, *Pilocarpus pennatifolius*, has a large latitudinal distribution as well as some of the greatest diversity of compounds present in the genus (Santos and Moreno, 2004; Sawaya et al., 2011). As *P. pennatifolius* has one of the largest distributions, one would expect that there would be a greater variation in chemistry since it is exposed to a larger variety of environmental factors. Using *P. pennatifolius* as an example of a species with a widespread latitudinal distribution, we were interested in determining which environmental factors are associated with the greatest variation among metabolomes of *P. pennatifolius* populations (Supplementary Figure 1). We hypothesize that there are patterns of chemistry, or chemoecotypes, correlated with varying environmental conditions among wild populations of *P. pennatifolius*.

Previous plant comparative metabolomics studies have utilized linear methods to investigate correlations between PSM and the environment (Kaplan et al., 2004; Arbona et al., 2013; Lankadurai et al., 2013; Sampaio et al., 2016). However, with the larger environmental gradient present in the wild populations of this study, the response of chemical variation in these situations follows a non-linear and unimodal distribution (Whittaker, 1956). In situations of smaller environmental gradients, a linear response could approximate the conditions well; however, when there is a unimodal response we expect that the linear model will not suffice (Šmilauer and Lepš, 2014). PSM production in plants can display a linear or unimodal response when faced with different abiotic factors. A unimodal response can display maximum compound abundance at an optima with low compound production at the extremes (e.g., no nutrient or excessive nutrient), or there can be maximum compound abundance at the extremes when a plant faces major environmental stressors (Supplementary Figure 2; Yan et al., 2004; Pennycooke et al., 2005; Albert et al., 2009; Ncube et al., 2012; Šmilauer and Lepš, 2014). Although unimodal methods can approximate linear responses well, the reverse is not true with linear methods. Therefore, in the case of our study with a large environmental gradient and a unimodal response of traits, only the unimodal method can accurately assess the response. Multivariate methods can address the issues related to unimodal distributions and have been used extensively in microbial ecology and community ecology, but they have not previously been applied to plant environmental metabolomics (Braak, 1986; Harborne and Jeffrey, 1993; Sardans et al., 2011; Paliy and Shankar, 2016).

The objective of our study was to test the hypothesis that there are correlations between chemotypes and environmental conditions for wild populations of *P. pennatifolius* using multivariate methods to analyze leaf metabolomics data. This included an unconstrained ordination analysis to assess overall variation in the metabolome among sites, a constrained ordination to assess metabolomic variation attributed to environmental predictors, and discriminant analysis to assess the group classifications for chemoecotypes of the various wild populations. To avoid inaccurate estimates from linear analysis, in this study we have used the following unimodal analyses, Correspondence Analysis (CA) (Hill, 1973, 1974) and Canonical Correspondence Analysis (CCA), to accurately assess variation in the metabolomes of wild populations of *P. pennatifolius*.

## Materials and Methods

### Field Collections of Plant Material and Plant Sampling Design

To examine variation in alkaloid and phenolic profiles due to environmental variation, this study focused on *P. pennatifolius*. This species was chosen because it has the largest latitudinal range (greater variation of climatic variables) that is both current and historic, and it is not threatened with extinction in natural populations. Using bioclimatic data from the WorldClim website^1^, distribution data from 2189 Brazilian herbarium specimens from the SpeciesLink website^2^, and the Ecocrop function in DIVA-GIS, localities with the greatest disparity of climatic variables were chosen for collection sites while planning our fieldwork itinerary (Dell et al., 2014).

Field collected plants were sampled from six locations for *P. pennatifolius*, obtaining as many individuals as possible from each site (Table 1). Specimens were collected from Parque Estadual Lago Azul, Campo Mourão Site 1, Campo Mourão Site 2, Foz do Iguaçu, Estação Ecológica St. Tereza, and Cruz do Pedro. Herbarium specimens were made for each site and can be accessed at the Bailey Hortorium Herbarium (BH) and USP Ribeirão Preto Herbarium (SPFR) (Supplementary Table 1 and Supplementary Figure 1). Multiple leaves were collected from each individual. Specifically, the third leaflet down from the terminal leaflet of the compound leaves was chosen; each sample collection was obtained from a different compound leaf each time.

**Table 1:** Summary of *P. pennatifolius* population sites and final set of environmental variables.

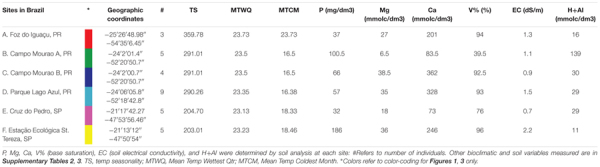

### Soil Sample Field Collections and Analysis

At each collection locality, two soil samples were collected at 20 cm depth (van Rai, 2001; Camargo et al., 2009) and soil analysis was carried out at the Instituto Agronômico in Campinas, SP, Brazil following established methods (van Rai, 2001). Fe, Mn, Zn, P. K, Ca, Mg, S, B, Na, Al, N, organic matter, pH, soil electrical conductivity (EC), Base saturation (V%), cation exchange capacity (CEC) were determined in the soil samples (Supplementary Table 2).

### Bioclimatic Data Retrieval

Bioclimatic variables (BIO1-BIO19) at 30-arc seconds were extracted from the WorldClim website (see text footnote 1) using the longitude and latitude coordinates of each collection locality. In addition, altitude data was extracted using coordinates for each collection on the program DIVA-GIS (Supplementary Table 3).

### Biochemical Extraction of Leaf Tissues for LCMS

To validate biochemical extractions of alkaloids and coumarins from field collected leaf tissue, a degradation study on two live specimens of *P. pennatifolius* from the New York Botanic Garden was conducted. Silica-dried leaf tissue on ice showed the least degradation and was not significantly different from the immediately frozen tissue. This method of preservation of leaf tissue for biochemical extractions has been used in many studies that require plant collections in remote areas, as the removal of water reduces enzymatic activity in the leaf tissue and the cold temperature helps to prevent enzymatic reactions due to handling (Fine et al., 2013).

Biochemical extractions to elucidate alkaloids and phenolics present in *Pilocarpus* leaf tissue were performed on silica-dried leaf tissue, at UNICAMP in Brazil. Alkaloid extraction protocols followed modifications made by Dr. Sawaya (Dr. Mazzafera Lab) on the method developed by Avancini et al. (2003). Alkaloid extracts were done on approximately 10 mg leaf tissue (normalized by exact weight in LCMS data prep). Leaf tissue was extracted with 1 ml of 0.1% Formic acid and placed in an ultrasonic water bath for 20 min. Supernatant was removed and 1 ml of 0.1% Formic acid step was redone twice, filtered, and analyzed. The phenolic extraction protocol followed the methods developed by Vialart et al. (2012) and Durand-Hulak et al. (2015). These phenolic extracts were done on approximately 200 mg tissue (normalized by exact weight in LCMS data prep). Tissue was homogenized in 2 ml 80% ethanol for 1 min, centrifuged for 10 min, and the supernatant removed to a fresh tube. Each individual plant had a total of three biological replicates for each extraction.

### UPLC-ESI-MS Methods

#### Alkaloids

Five microliters of each sample were injected into an Acquity UPLC coupled with a TQD triple-quadrupole mass spectrometer (Micromass-Waters, Manchester, United Kingdom). Mass spectrometer conditions were: capillary 3.0 kV, cone 30 V, extractor 1V, ion source temperature 150°C, desolvation temperature 300°C and column temperature 30°C, in electrospray ionization in positive mode. The chromatographic column used was a Polaris 3 C18-A 100 mm × 2.0 mm column (Varian) and the elution was carried out using an ammonium acetate buffer, 10 mmol/L, pH 3.0 (solvent A) and acetonitrile (solvent B) in a gradient ranging from 5 to 25% of solvent B in 8 min. The retention time and m/z were used to identify alkaloid compounds in the samples.

#### Phenolics

Sample extracts were diluted in 80% ethanol (ethanol and water) 1:4. Four microliters of each sample were injected into an Acquity UPLC coupled with a TQD triple-quadrupole mass spectrometer (Micromass-Waters, Manchester, United Kingdom). Mass spectrometer conditions were: capillary 3.0 kV, cone 35 V, extractor 1V, ion source temperature 120°C, desolvation temperature 350°C and column temperature 30°C, in electrospray ionization in positive mode. The chromatographic column used was a Waters ACQUITY C18-BEH (2.1 mm × 100 mm, 1.7 μm) column and the elution was carried out using Milli-Q water with 0.1% formic acid (solvent A) and acetonitrile (solvent B) in a gradient ranging from 10 to 100% of solvent B in 8 min. The retention time and m/z were used to identify coumarin compounds in the samples using a SIM (single ion monitoring) mode. For the metabolomics analysis samples were run in TIC (total ion chromatogram) mode for all phenolics.

### LCMS Data Processing

Data processing was conducted using LCMS raw data in MZmine2 (Pluskal et al., 2010) and XCMS in R (Smith et al., 2006). Waters raw LCMS data were converted into mzXML format using the MSConvert package in Proteowizard v.3.0.10051 (Chambers et al., 2012). The raw data in mzXML format was then processed in MZmine2 following protocols described by Earll (2012): mass detection (*m/z* tolerance of 0.002) and filtering, chromatogram builder, peak deconvolution using Wavelets XCMS algorithm, peak alignment of all the LCMS runs, adduct search, and gap filling (Earll, 2012). The raw data was also analyzed using XCMS in R, this included peak picking, integration, and non-linear retention time correction of peaks between samples. LCMS data of all the metabolite features were exported, including EIC (extracted ion chromatograms) for manual analysis and a csv file of the processed samples containing: peak *m/z*, peak RT, and peak intensity areas for multivariate analyses. Some compounds were identified via standard references as well as comparison with literature (Sawaya et al., 2011; Hiserodt and Chen, 2012; Tine et al., 2017; Supplementary Tables 4A,B). All metabolite features used in the analysis are in Supplementary Table 5A (alkaloid extract) and Supplementary Table 5B (phenolic extract). LCMS data was normalized by dry weight of leaf tissue, log transformed, and pareto scaled to analyze the metabolome of individuals from these six sites.

### LCMS Multivariate Analysis

Unsupervised multivariate methods, CA and HCA were employed in CANOCO 5. CA was used to reduce the dimensionality of the LCMS data. Hierarchical cluster analysis (HCA) was utilized to create a dendrogram, utilizing Ward’s method with Euclidean distances to confirm affinities from CA.

Constrained ordination is not possible with 39 environmental predictors, as the result will be unconstrained; therefore, the 39 environmental predictors were reduced to eleven environmental predictors, to reduce multicollinearity and the arch effect due to many environmental variables (Draper and Smith, 1998; Šmilauer and Lepš, 2014). Factor analysis of the environmental variables revealed five components for the 39 environmental predictors. These complex variables were made up of many environmental predictors and the variables did not load clearly; consequently, the interpretability would be lost if these complex variables were used in the constrained ordination (Draper and Smith, 1998; Šmilauer and Lepš, 2014). Evaluation of the sites on Canoco software revealed environmental variables with low contributions/arrows, which were removed first from the data set. Forward stepwise analysis was performed on Canoco software using a semi-automated procedure, determining the eleven most informative variables that explained the residual variation in chemical traits. This stepwise analysis also analyzed correlated variables, assessing the difference in eigenvalues of species-environmental correlations to determine if wrong variables or too many variables were removed from correlated sets. The final set of included variables is presented in Table 1; however, for both analyses only four variables had significant contributions to the chemical variation.

Canonical Correspondence Analysis of environmental predictors was used to assess variation of metabolic profiles, associated with environmental variables. The three environmental variables with greatest significant variation affecting profiles were depicted on CCA.

Supervised multivariate analyses such as Partial Least Squares Discriminant Analysis (PLS-DA) using the R pls package through MetaboAnalyst, and Random Forest analysis using the randomForest package in R through MetaboAnalyst, were used to confirm classification of chemotype groups (Xia and Wishart, 2002). PLS-DA is excellent at managing both noisy data and multicollinearity, and it was used to identify significant variables in chemotypes through the determination of the regression coefficient, loading plots and variable importance on projection (VIP). Random Forest Analysis was also used for class prediction and provides error rates for each class as well as outlier measures, OOB error, and variable importance measures.

### Validation of Multivariate Analyses

The significance of the class discriminations described by PLS-DA models were statistically validated using performance accuracy measures R2 (sum of squares captured by the model) and Q2 (cross-validated R2), cross-validation with different numbers of components and a permutation test using the optimal number of components from the cross-validation.

## Results

### *Pilocarpus pennatifolius* Sample Sites and Environmental Variables

The choice of environmental variables to use in the model can affect the analysis; therefore, forward selection of environmental predictors in Canoco was used to reduce the environmental variables for the analysis (Table 1). Leaf tissue underwent two separate extraction protocols, an alkaloid and a phenolic extraction, and was normalized by dry weight of leaf tissue and log transformed, to analyze the metabolome of individuals from these six sites. All identified and unidentified peaks were used for the metabolomic analyses (Supplementary Tables 5A,B).

### Correspondence Analysis to Assess Overall Variation Among Sites

The relationship between the compound abundance across samples, versus the measured values of the environmental variables was evaluated. As many of our environmental variables encompass a broad range, we analyzed the distribution in SPSS and found that very few variables followed a linear response; therefore, it was preferable to use unimodal analysis methods for our analysis (Šmilauer and Lepš, 2014). For our indirect gradient analysis we used the unimodal method of correspondence analysis.

**FIGURE 1:**
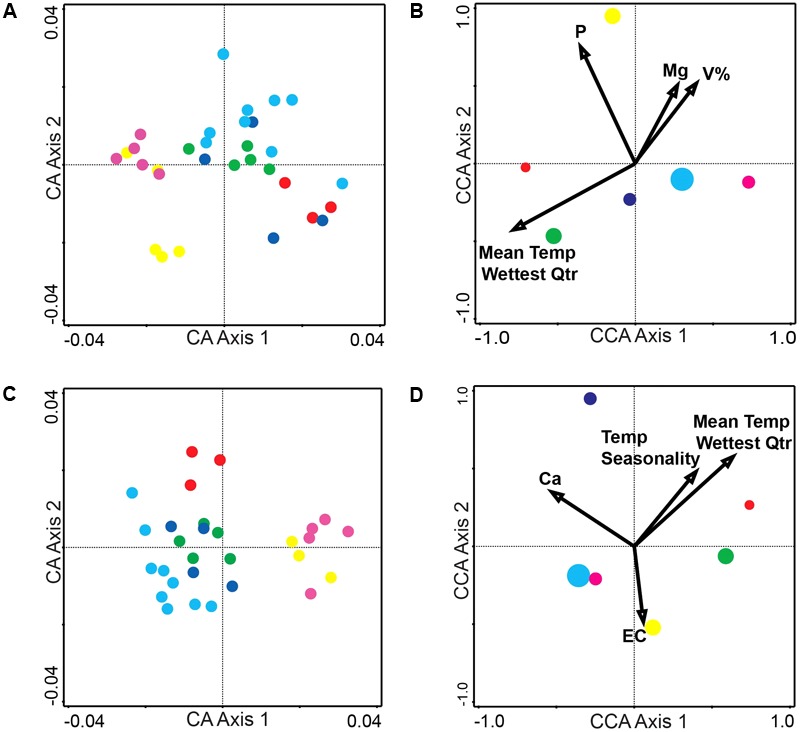
Correspondence Analysis (CA) and Canonical Correspondence Analysis (CCA) for the alkaloid and phenolic extractions. **(A)** CA ordination plot of alkaloid extraction at the six sites with each dot representing an individual plant (average of three biological replicates) at a site **(B)** CCA ordination biplot of alkaloid extractions displaying environmental variables (arrows) and each site (circle with size representing the number of individuals collected) **(C)** CA ordination plot of phenolic extraction at the six sites with each dot representing an individual plant (average of three biological replicates) at a site **(D)** CCA ordination biplot of phenolic extraction displaying environmental variables (arrows) and each site (circle with size representing the number of individuals collected). Number of individuals collected and site location (A–F) is also noted in Table 1. Numerical results of CA and CCA are in Table 2. Site A is Red, Site B is Green, Site C is Dark Blue, Site D is Light Blue, Site E is Magenta, and Site F is Yellow.

Correspondence analysis, also known as reciprocal averaging, is based on the weighted averaging of compound scores to estimate the latent variables that best predict values for all compounds in a model (Lepš and Šmilauer, 2003). CA of metabolomics data from the populations of the six sites depicts about four major groups of variation in the alkaloid extract (Figure 1A) and about three major groups of variation in the phenolic extract (Figure 1C). The explained variation of each axis of the CA ordination is shown in Table 2. The first few axes of CA are usually attributed to environmental variables; however, as this is a secondary comparison to environmental gradients, it is an indirect gradient analysis and is viewed as the overall chemical variation between samples. When each compound is graphed on the CA diagram we can see how each grouping of samples is differentiated by a greater abundance of select compounds in the chemical profile (Supplementary Figures 3A,B).

**Table 2:** Explained variation from axes in the CA and CCA ordination diagrams.

	Axis 1	Axis 2	Axis 3	Axis 4
**Alkaloid extraction**				
Unconstrained (CA) explained variation (cumulative)	30.95	47.65	58.04	65.61
Constrained (CCA) explained variation (cumulative)	11.23	18.14	21.41	23.33
Pseudo-canonical correlation	0.629	0.715	0.658	0.586
**Phenolic extraction**				
Unconstrained (CA) explained variation (cumulative)	29.09	41.28	50.48	58.84
Constrained (CCA) explained variation (cumulative)	13.98	20.47	24.70	27.83
Pseudo-canonical correlation	0.751	0.845	0.746	0.649

### Canonical Correspondence Analysis to Assess Variation Due to Environmental Factors

To directly test the environmental gradient, CCA, a multivariate extension of CA was applied. Here the axes are restricted or constrained to be linear combinations of environmental variables, and therefore the axes represent those variables that present the maximum separation or variation in the samples. The total environmental variables collected for each site consisted of 19 bioclimatic variables from the WorldClim website (see text footnote 1) and 20 soil variables analyzed from soil samples at each site. Using all 39 environmental variables for the CCA would create an ordination diagram that appears more comparable to an unconstrained CA than to a constrained CCA. Therefore, after removing collinear variables we performed a forward selection analysis of explanatory variables in CANOCO, to test which variables would improve the fit (Tables 1, 3). The CCA ordination diagram of the alkaloid extraction depicts the four most important variables contributing to the chemical variation between the sites as: mean temperature of wettest quarter as well as the content of phosphorus, magnesium, and V% – base saturation in the soil (Figure 1B). The CCA ordination diagram of the phenolic extraction depicts the four most important variables contributing to this chemical variation between the sites as: mean temperature of the wettest quarter, temperature seasonality, calcium, and soil EC (Figure 1D). The explained variation of each axis of the CCA ordination diagrams is shown in Table 2. Next, we tested the constrained axes to verify the significance of the axes: the first and second axes were significant for the alkaloid extract, whereas only the first axis was significant for the phenolic extracts (Table 4).

**Table 3:** Interactive forward selection of environmental variables.

	Explains %	Contribution %	*P*-value
**Alkaloid extraction**			
Mean Temp Wettest Qtr.	11.1	35.4	0.008
P	8.9	28.4	0.018
V% (Base Saturation)	4.5	14.4	0.377
Mg	3.5	11.1	0.664
**Phenolic extraction**			
Mean Temp Wettest Qtr.	8.5	28.6	0.032
Ca	7.4	24.8	0.073
EC (Soil Electrical Conductivity)	6.0	20.1	0.167
Temp seasonality	4.7	15.8	0.397

**Table 4:** Significance of constrained axes of CCA diagrams.

	Axis 1	Axis 2	Axis 3	Axis 4
**Alkaloid extraction**				
Explained by constrained axis	14.71%	8.60%	3.06%	1.53%
*P*-value	0.007	0.03	0.68	0.894
**Phenolic extraction**				
Explained by constrained axis	13.95%	6.48%	4.21%	1.86%
*P*-value	0.013	0.163	0.4	0.867

**FIGURE 2:**
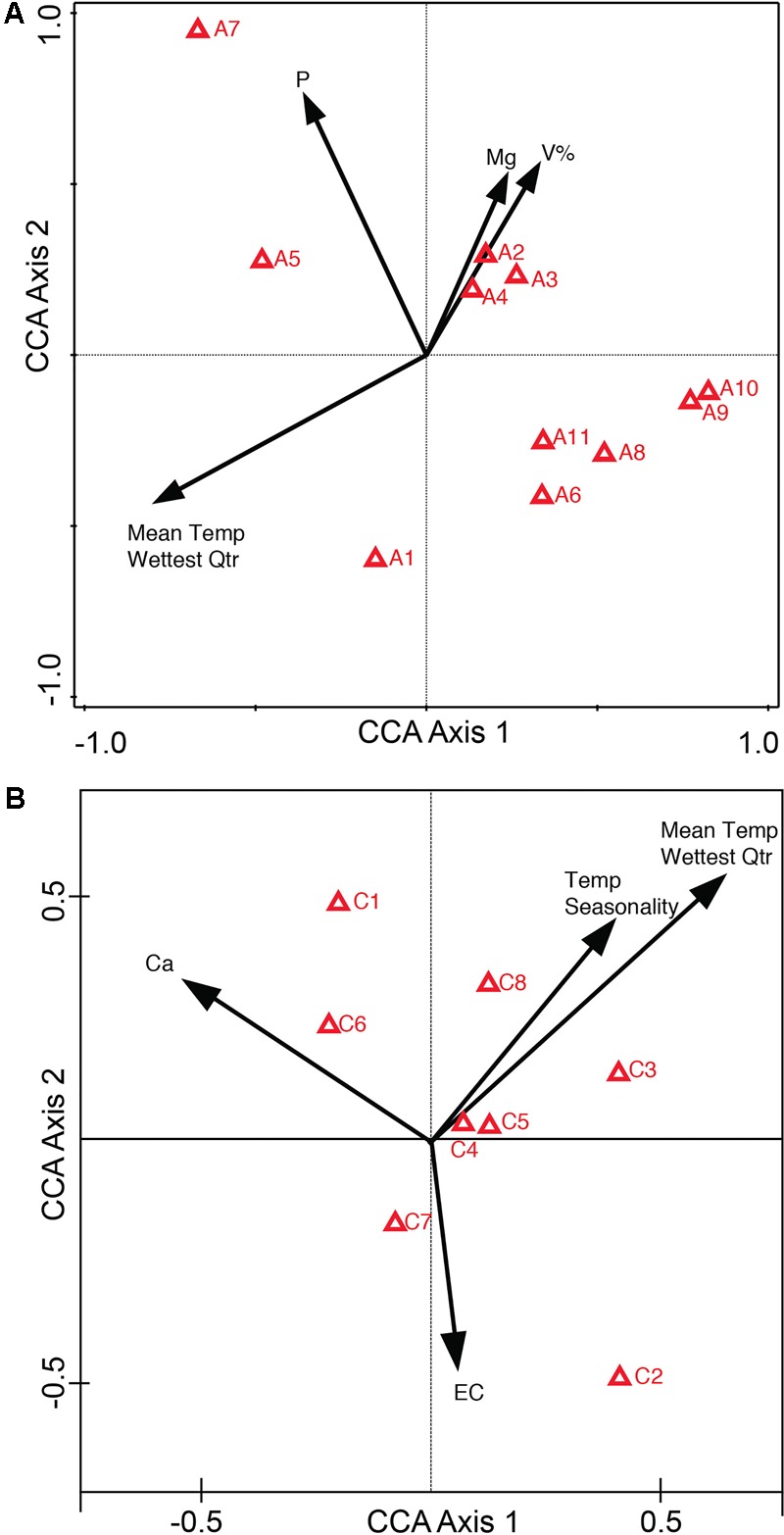
Compounds-environmental variables biplot of Canonical Correspondence Analysis (CCA) for identified compounds. CCA biplot depicts environmental variables as arrows and compound optima as red triangles (IDs refer to Supplementary Tables 4, 6). From the ordination one can determine how higher levels of environmental variables affect the abundance of the compound. Compound A7’s optima, or the highest abundance of A7, occurs at higher levels of soil P, whereas compound A6 has a higher compound abundance with low soil P **(A)** CCA of alkaloid compounds (A1–A11) **(B)** CCA of coumarin compounds (C1–C8).

Canonical Correspondence Analysis ordination diagrams can also be plotted as a biplot of the response variables (compounds) and the explanatory variables (environmental variables). The compounds-environmental variables CCA biplot of the entire metabolome (Supplementary Figure 4A: Conceptual Diagram, Supplementary Figure 4B: Alkaloid extraction, and Supplementary Figure 4C: Coumarin extraction) depicts how different groups of the metabolome are affected by higher and lower levels of the environmental variables present across the collection sites. Next, the compound optima (red triangles) in the compounds-environmental CCA biplot (Supplementary Figure 4) were reduced to depict only the identified compounds. This was done to analyze how higher/lower levels of environmental variables across the sites affect the abundance of these specific compounds (Figure 2A: Alkaloids, Figure 2B: Coumarins). Supplementary Table 6 is provided to summarize the levels of environmental variables that have been associated with abundance optima of the following identified compounds.

In the Alkaloid CCA biplot (Figure 2A), compounds A7 [3-Hydroxymethyl-4-(3-methyl-3H-imidazol-4-yl)-1-phenylbutan-1-one] and A6 [3-(Hydroxy-phenyl-methyl)-4-(3H-imidazol-4-ylmethyl)-dihydro-furan-2-one] illustrate high compound abundance at opposite levels of environmental variables. A7 has a higher abundance in the sites with higher soil P, Mg, V%, as well as mid-to-high values of Mean Temperature in the Wettest Quarter. A6 on the other hand has a higher abundance in the sites with lower soil P, Mg, V% as well as lower values of Mean Temperature in the Wettest Quarter. The abundance optimum of pilocarpine (A4) appears to be associated with the same environmental variables as the optima of two other identified compounds: A3 (pilocarpidine) and A2 [4-(3H-imidazol-4-ylmethyl)-3-methyl-5H-furan-2-one]. These three compounds have greater compound abundances when present at sites with high levels of soil Mg and V% (Base Saturation), mid-to-high levels of soil P, and low Mean Temperature in the Wettest Quarter. The CCA biplot of the coumarin compounds (Figure 2B) shows less indication of groupings. C1 (scopoletin) and C2 (psoralen) appear to have the most distinct response to environmental variables. C1 has its highest abundance at sites with high soil Ca, high temperature seasonality, high Mean Temperature in Wettest Quarter, and low soil EC. However, the opposite is true for C2, where the highest abundance of C2 is associated with the sites that have low soil Ca, low Temperature Seasonality, low Mean Temperature in the Wettest Quarter and high soil EC.

### Classification of Chemoecotypes

**FIGURE 3:**
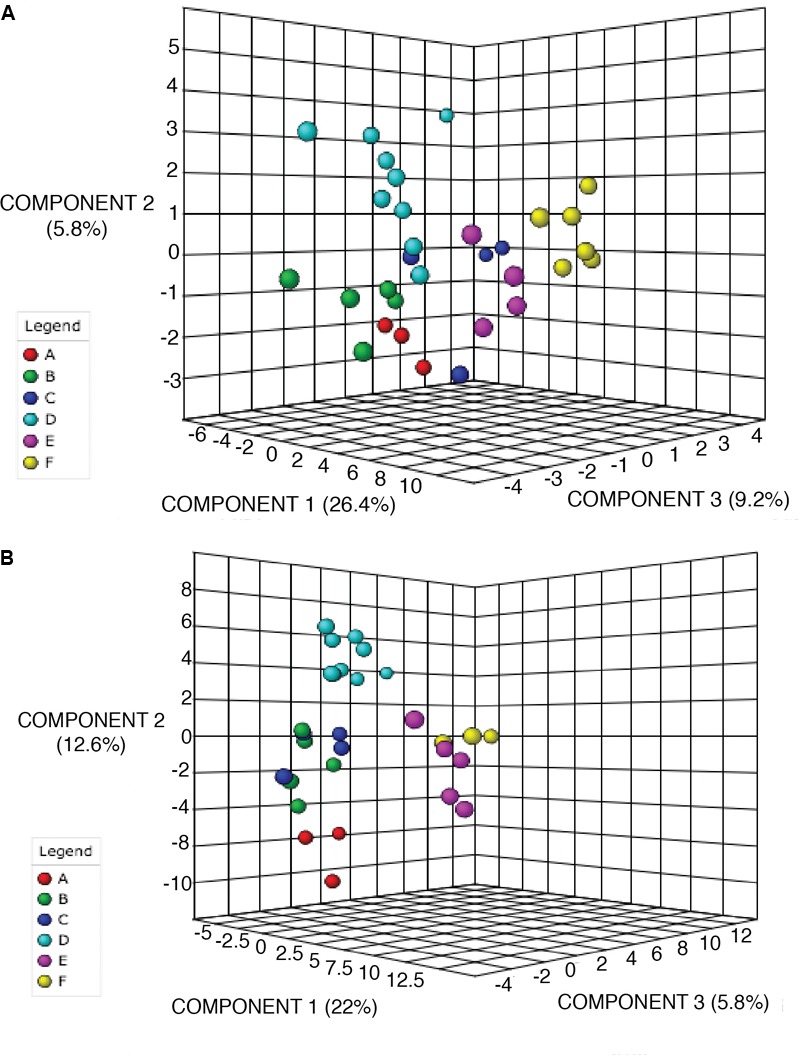
PLS-DA of alkaloid and phenolic extractions depicts differentiation between sites. 3D scores plot between selected PCs. The explained variances are shown in brackets. Site locations (A–F) are color-coded and ID’s refer to Table 1. **(A)** PLS-DA for alkaloid extraction **(B)** PLS-DA for phenolic extraction.

To further confirm the classification of chemoecotypes by site we used Partial Least Squares-Discriminant Analysis and Random Forests Analysis. PLS-DA was used on the log-transformed LCMS data. Possible hypotheses for the chemical variation at sites include: no variation between sites leading to one chemotype for all sites (Supplementary Figure 5A), visible groupings or chemoecotypes identified per site (Supplementary Figure 5B), or random chemical variation within and among sites (Supplementary Figure 5C). For both alkaloid and phenolic extractions we were able to confirm group classifications/chemoecotypes for each site. For the alkaloid extraction, we note a clear grouping of individuals from each site in the PLS-DA 3-D scores plot, though we note that Site C has 1 outlier (Figure 3A). In the phenolic extraction, we also see a grouping of individuals from each site on the PLS-DA 3-D scores plot (Figure 3). PLS-DA 3-D scores plot of all biological replicates at all sites was also completed, and a dense grouping at each site is noted (Supplementary Figures 6A,B). Statistical validations of the class separations of PLS-DA were done through the cross-validation of different numbers of components and permutation tests of randomly assigned class labels using the optimal number of components (Supplementary Figure 7 and Supplementary Table 7).

**FIGURE 4:**
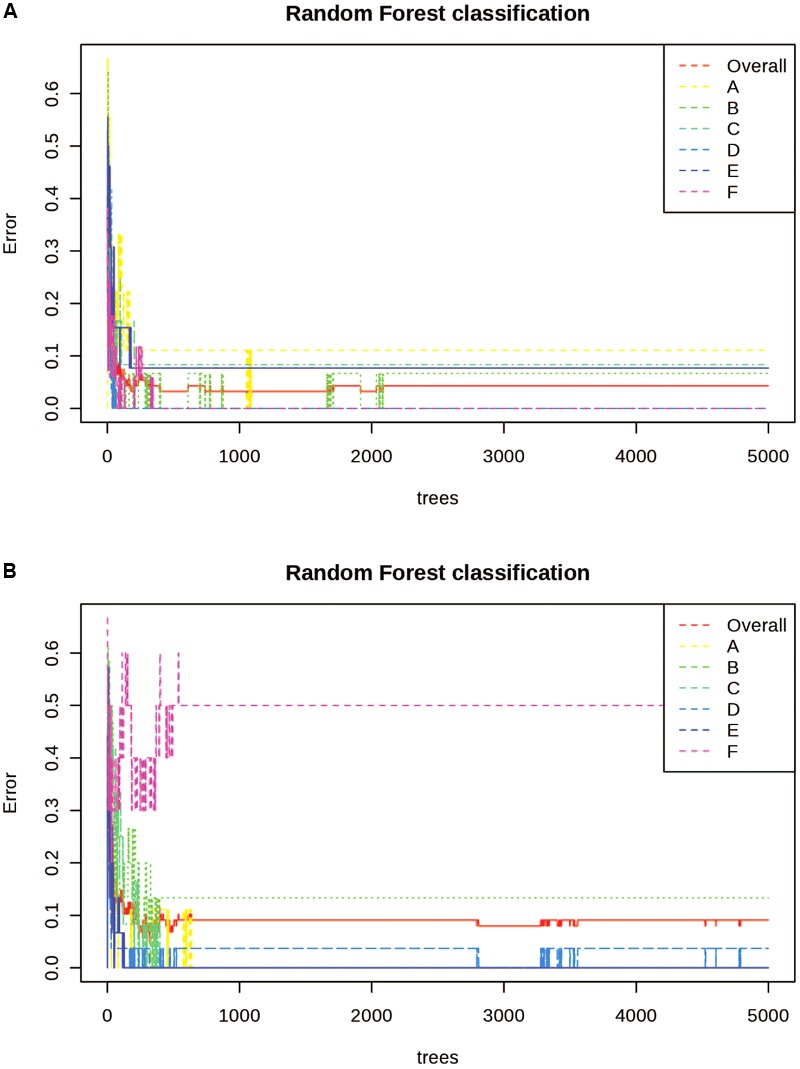
Random Forests (RF) Classification of the differentiation between sites for alkaloid and phenolic extractions. Each color represents the cumulative error rates for the RF classification of each site as a unique class/chemoecotype. A site with a smaller error rate that is stabilized with a fewer number of decision trees is considered to be more accurately distinguished as a unique chemoecotype. The overall error rate for chemoecotype classification is shown as a red line. Site locations are color coded by the legend on this graph and labeled with ID’s (A–F) as found on Table 1. The alkaloid extraction has the lowest overall error rate for classification of chemoecotypes, therefore chemoecotype groups are more easily distinguished or identified. **(A)** alkaloid extraction **(B)** phenolic extraction.

Random Forest Analysis was also utilized for class prediction. In Figure 4A,B we see that the classification of sites based on the alkaloid extraction (Figure 4A) is more defined than the phenolic extraction (Figure 4B). This is also confirmed by the OOB error and error rates for each class seen in Table 5. The variation in certain groups or sites could be due to sample size; however, it is not possible to acquire many individuals of this species at every location.

**Table 5:** Random Forests classification performance matrix.

	A	B	C	D	E	F	Class error
Alkaloid extraction: OOB Error = 0.0323							
**A**	9	0	0	0	0	0	0
**B**	0	14	0	1	0	0	0.0667
**C**	0	0	11	1	0	0	0.0833
**D**	0	0	0	27	0	0	0
**E**	0	0	0	0	12	1	0.0769
**F**	0	0	0	0	0	17	0
Phenolic extraction: OOB Error = 0.0909							
**A**	9	0	0	0	0	0	0
**B**	0	13	0	2	0	0	0.133
**C**	0	0	12	0	0	0	0
**D**	0	0	1	26	0	0	0.037
**E**	0	0	0	0	15	0	0
**F**	0	1	0	0	4	5	0.5

## Discussion

Just as it is possible to determine species growing in an area based on environmental conditions present at a location (Braak, 1986; Harbert and Nixon, 2015), it is also possible to predict which compounds or compound profiles will be more abundant at environmental optima through the use of direct gradient analyses. Metabolomic expression across the distribution of a plant species can follow a non-linear, non-monotonic relationship with environmental variables, and this can be verified in situations of large environmental gradients (Whittaker, 1956). To accurately analyze non-linear responses, unimodal methods of ordination diagrams are preferred.

In our study, we analyzed the metabolite profiles of *P. pennatifolius* populations using two extraction protocols, alkaloid and phenolic. For the alkaloid extract, we found that the most significant environmental variables contributing to the variation in alkaloid profiles were mean temperature of the wettest quarter, as well as the soil content of phosphorus, magnesium, and V% (base saturation). Temperature and precipitation are known to affect alkaloid content in plants, and thus variations in these bioclimatic factors could provide variations in chemical profiles (Waller, 1978; Harborne and Jeffrey, 1993). It is usually expected that nitrogen availability has a strong effect on alkaloid production as alkaloids are N containing compounds, but this is not always true. Previous stress studies irrigating nutrient solutions on *P. microphyllus* seedlings found that N omission in nutrient solutions did not affect pilocarpine production as much as K omission, as is further shown in our study (Avancini et al., 2003). This low response could be due to a reduced availability of nitrogen in an accessible form or other limiting nutrients that are needed in conjunction with nitrogen for a balanced nutrition. Base saturation (V%) indicates the amount of basic cations in the soil, and is essential for determining soil fertility as it specifies the percentage of sodium, calcium, potassium, and magnesium that is a part of the CEC. Sites A and B have high CEC values (250.50 and 232.05); however, their respective V% values (94 and 39.5%) demonstrate the great difference in fertility of these soils. The poor soil fertility of Site B is also verified by the lower pH and higher H+Al values. Minerals such as phosphorus and magnesium are very important in various enzymatic processes throughout the plant, and they have large roles in promoting plant development and growth. Studies examining the effect of increases in phosphorous and magnesium have had contradictory results in regards to the effect on alkaloids, ranging from no effect to both positive and negative effects (Waller, 1978; Bramble and Graves, 1991; Mazzafera, 1999). A study that examined the effect of Phosphorus omission on *P. microphyllus* found no effect on the pilocarpine content; however, the authors did not look at the effect on the overall alkaloid profile (Avancini et al., 2003; Abreu et al., 2005).

The most significant environmental variables contributing to the variation in the phenolic profiles in the phenolic extracts were mean temperature in the wettest quarter, temperature seasonality, as well as the soil content of calcium and the soil EC. Temperature seasonality has been known to have a strong effect on compound expression, since areas of greater seasonality are faced with more variation and could lead to greater variation in chemistry (Molina-Montenegro and Naya, 2012). Specifically, work on the synthesis of phenolics has found that they are regulated not only by developmental signals, but also signals from the environment (Figueiredo et al., 2008; Carbone et al., 2009; Jaakola, 2013). The potential role of calcium is vast as it is essential for membrane permeability, in addition to being a messenger for pathways in development, responses to environmental stimuli and in response to plant defense (Hepler, 2005; Lecourieux et al., 2006; Zhang et al., 2014). In fact, various studies have shown that calcium treatment has led to an increase in phenolics, due to the upregulation of important genes in phenolic compound metabolism (Xu et al., 2014; Zhang et al., 2014; Ahmad et al., 2016). On the other hand, it appears that Phenylalanine ammonia-lyase (PAL), the first enzyme of the phenylpropanoid biosynthetic pathway, is negatively affected by high concentrations of calcium; therefore, it is likely that a variation in calcium could affect the phenolic profile in *P. pennatifolius* (Chishaki and Horiguchi, 1997; Tomás-Barberán et al., 1997; Teixeira et al., 2006; Xu et al., 2014). PAL is indispensible for the synthesis of a range of metabolites, and it has been found that PAL activity also varies when faced with environmental stimuli such as thermal stress (Christie et al., 1994; Hunter et al., 1996; Rivero et al., 2001; Payyavula et al., 2012). Soil EC is also important for plant defense and stress management. EC is the ability of the soil to conduct electricity, and it is determined by a variety of factors including soil moisture, clay content, soil temperature and salinity (including salts such as Na, Ca, K, Mg, Cl, SO4, CO3) (Hanlon, 1993). Various studies have found that higher soil conductivity can indicate greater fertility when there is a higher CEC or a higher sum of bases (Ca, Mg, and K) (Fraisse et al., 2001; Officer et al., 2004; Moral et al., 2010). This greater availability of nutrients can increase the fertility of the soil, thereby promoting growth and subsequently metabolites produced, or it could also present a decrease in metabolites as per the growth-defense trade-off (Coley et al., 1985; Herms and Mattson, 1992; Hunter et al., 1996; Fine et al., 2006; Endara and Coley, 2011). In this study the sum of bases is highly positively correlated with CEC (Pearson’s *r* = 0.94); however, EC has a moderately positive correlation with CEC (Pearson’s *r* = 0.28) and EC has a moderately positive correlation with the sum of bases (Pearson’s *r* = 0.34). As the correlation of EC and CEC is weak, it is possible that other factors are affecting the value of EC in these soil samples. These factors could be related to soil moisture/temperature or the clay soil type present, which in combination can affect the soil microbiome, thus affecting the phenolic profile present (Sudduth et al., 2003; Badri et al., 2013). Further work is required to assess soil texture and water drainage contributions to the EC values at these sites.

There are many factors that can affect a plant’s metabolome and the response of each plant depends on its genetic profile, its environment, as well as the interaction between genotype and the environment. Plasticity is the term used to describe how one genotype can have multiple phenotypes, depending on the environment (Thoday, 1953). Local adaptation depends on both genetic and environmental factors to determine phenotypes with the greatest fitness; however, there is a gradient of importance for each quality (Spichtig and Kawecki, 2004). Several studies have found that areas of stable environmental factors, such as constant temperatures (i.e., the tropics), are correlated with reduced plasticity of PSM (Sultan, 1987; Molina-Montenegro and Naya, 2012; Tack et al., 2012; Gratani, 2014). As plants are immobile, variation in abiotic and biotic interactions leads to an adaptation to the environment, through the modification of a plant’s chemical defense. Consequently, stability in the environment would reduce the overall stresses facing the plant, decreasing the selective forces favoring plasticity of the plant to change its metabolite phenotype for survival, which reduces its metabolite variation. There are many instances of a single plant species having different phytochemical profiles in different locations (Hunter et al., 1996; Koricheva, 1999; Andrade-Neto et al., 2002; Hu et al., 2007; Endara and Coley, 2011; O’Reilly-Wapstra et al., 2013; Moore et al., 2014; Moustafa et al., 2016). This could be due to different chemotypes (distinct genetically determined phytochemical profiles that are not evolutionary plastic), plasticity of a single genotype, or gradients of plasticity with genetic variance (Santesson, 1968; Keefover-Ring et al., 2009; Gratani, 2014). Determining the plasticity of a species is difficult without a common garden or transplant experiment.

As our study used only ordination methods to associate environmental variables with variation in chemical profiles of *P. pennatifolius*, and correlation does not imply causation, there are still many laboratory and greenhouse experiments needed, such as seedling stress tests that can be done to further confirm the effect of environmental variables on the chemical profile. In addition, further identification of the compounds found to be significantly different between chemoecotypes through CA, CCA, PLS-DA, and RF could lead to a more targeted analysis and understanding of biological pathways. Common garden experiments, planting different genotypes in the same location, could also be very useful to detect whether differences in chemical variation are indeed due to environmental factors or genetic differences. Either result would be beneficial for breeding as one could breed individuals for specific genes, or one could add/omit nutrients and stressors to modify compound yields. In the case of *P. pennatifolius*, a rare and slow growing tree, we are unable to run a common garden experiment. Therefore this exploratory analysis in wild populations is advantageous and essential as it has reduced the environmental variables, and will allow for a more guided experimental analysis.

## Conclusion

Analysis of wild populations of *P. pennatifolius* has provided us with potential environmental variables that should be followed up with greenhouse and in-field experiments to determine their importance in alkaloid and phenolic biosynthesis. Of course, the expression of compound profiles in plants is related to a variety of conditions, plant-environment interactions, plant genotype and genotype-environment interactions. Future work in our laboratory will examine population genetic differences among the various sites to assess variation that can be attributed to specific genotypes.

As the field of metabolomics is expanding into larger ecological studies examining wild populations, it is important to determine the best way to assess the response curves of compound profiles present in samples of a large population gradient. As there are non-monotonic and non-linear relationships between compound profiles and environmental variables, the use of unimodal methods (CA, CCA) instead of linear methods (PCA, RDA) will be much more accurate to describe the variation in ecometabolomic studies. As such our study has used exploratory data analysis methods common in community and microbial ecology studies, to perform an ecological metabolomics study of wild populations of *P. pennatifolius*. These unimodal methods can also be used in studies involving analysis of morphology, behavior, pollination, or physiological responses in wild populations across large gradients.

## Data Availability

All datasets generated for this study are included in the manuscript and/or the Supplementary Files.

## Author Contributions

DA conceived and designed the project, performed most of the experiments, conducted and interpreted the analysis with contributions from all of the authors, and wrote the article with contributions from all the authors. PM and KN supervised the experiments. EK conducted and performed the LCMS runs. KN supervised and complemented the writing.

## Conflict of Interest Statement

The authors declare that the research was conducted in the absence of any commercial or financial relationships that could be construed as a potential conflict of interest.

## Footnotes

1 www.worldclim.org

2 http://splink.cria.org.br
