# Supplementary material for: Ecometabolomic Analysis of Wild Populations of Pilocarpus pennatifolius (Rutaceae) Using Unimodal Analyses
Source: Front Plant Sci. 2019 Mar 6;10:258. doi: 10.3389/fpls.2019.00258 (PMC6414451; doi:10.3389/fpls.2019.00258)
Supplement: Supplementary file 1 [file Data_Sheet_1.pdf]

Supplemental materials

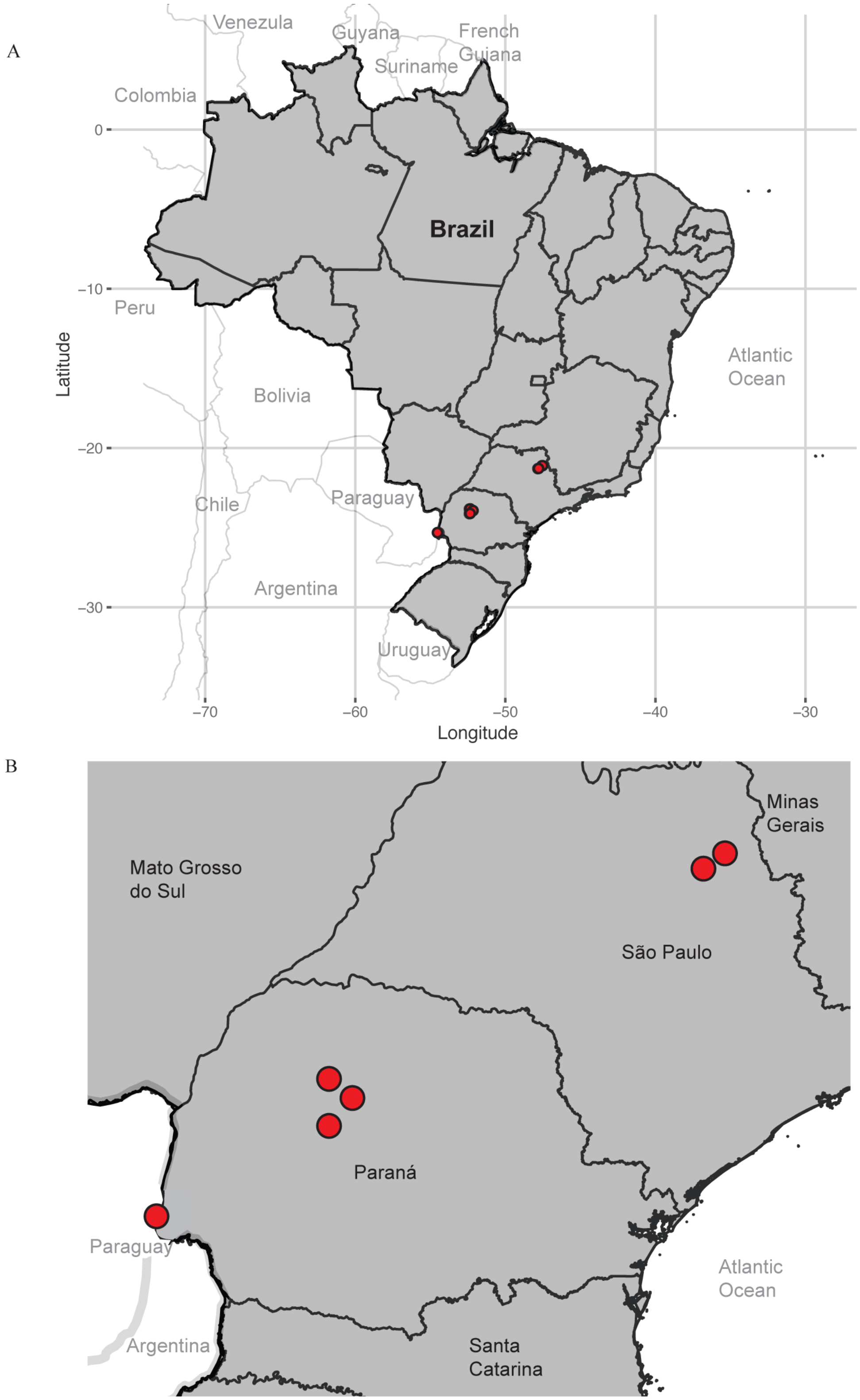

Supplementary Figure 1. Collection Localities of *Pilocarpus pennatifolius*. (a) Localities collected across Brazil (b) Magnified state-view of plant collections.

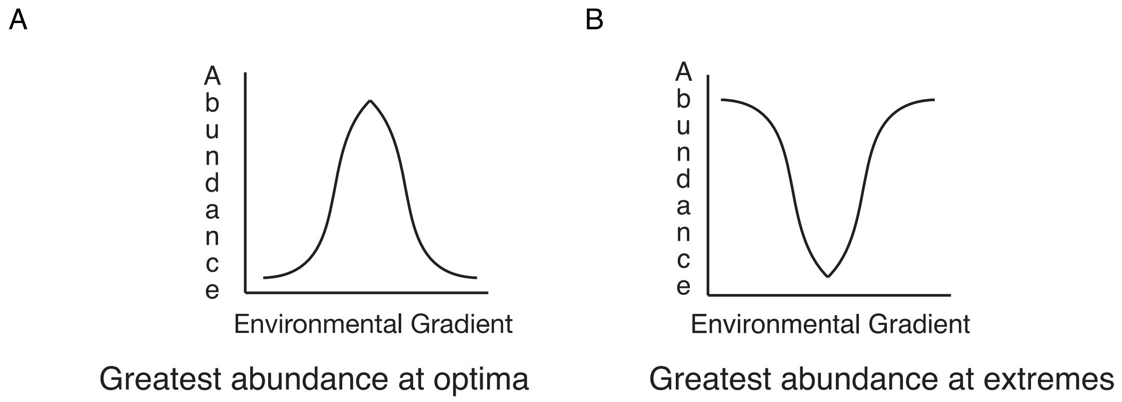

Supplementary Figure 2. Compound abundance can follow a unimodal response with regard to environmental factors. Greatest abundance at an optima (a), or greatest abundance at environmental extremes when the plant is under stress (b).

A

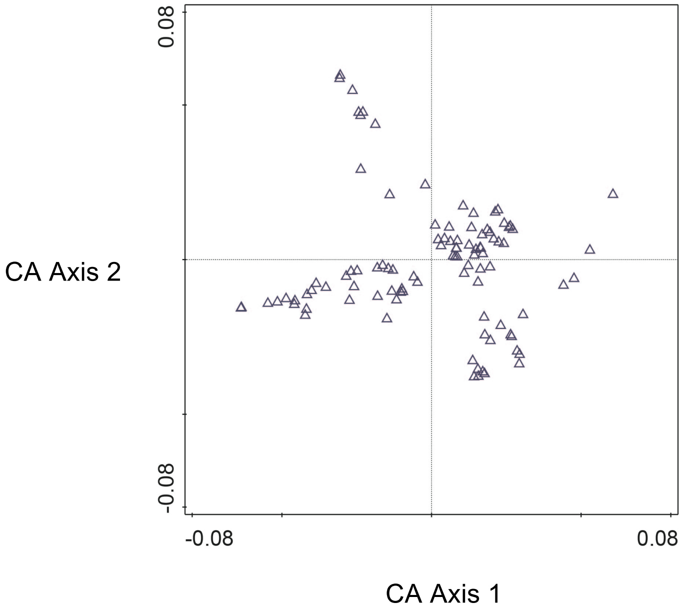

B

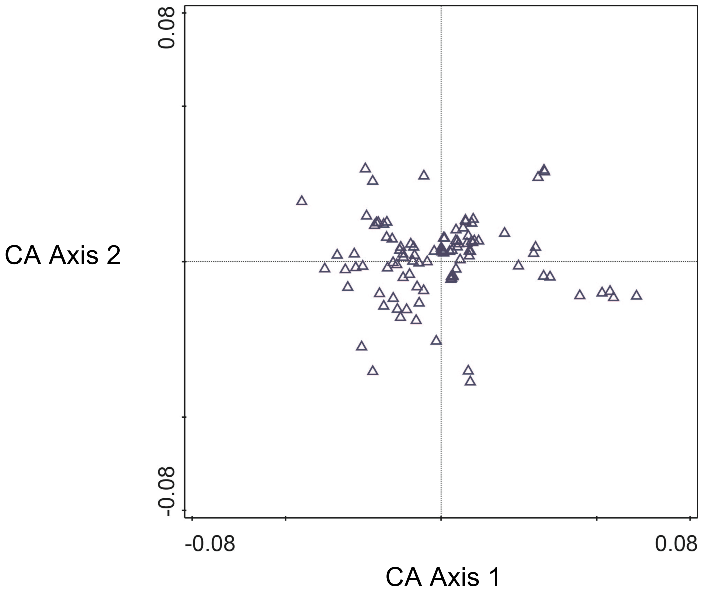

Supplementary Figure 3. Correspondence Analysis (CA) depicting compound optima. This plot depicts the variation in compounds across the six sites. Each triangle represents the optimum of each compound. (a) alkaloid extraction (b) phenolic extraction

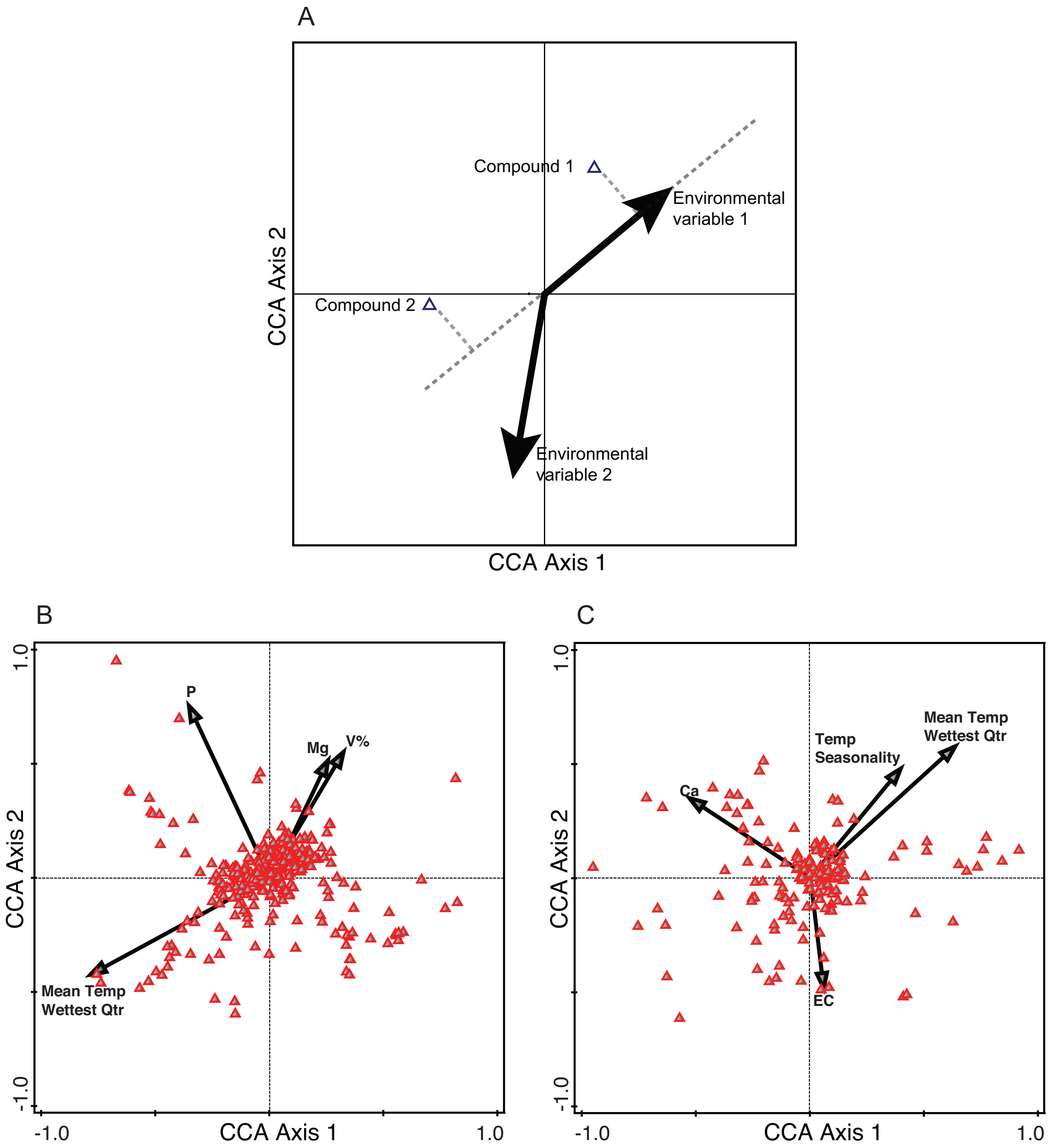

Supplementary Figure 4. Compounds-environmental variables biplot of Canonical Correspondence Analysis (CCA) (a) *Conceptual Diagram*: Compound optima (depicted as triangles) can be projected to the arrows of environmental variables. The optima/greater abundance of Compound 1 is present at higher values of Environmental variable 1, when compared with the optima of Compound 2 (b) CCA depicting environmental variables chosen by forward selection with greatest effect on the alkaloid metabolome (c) CCA depicting environmental variables chosen by forward selection with greatest effect on the coumarin metabolome.

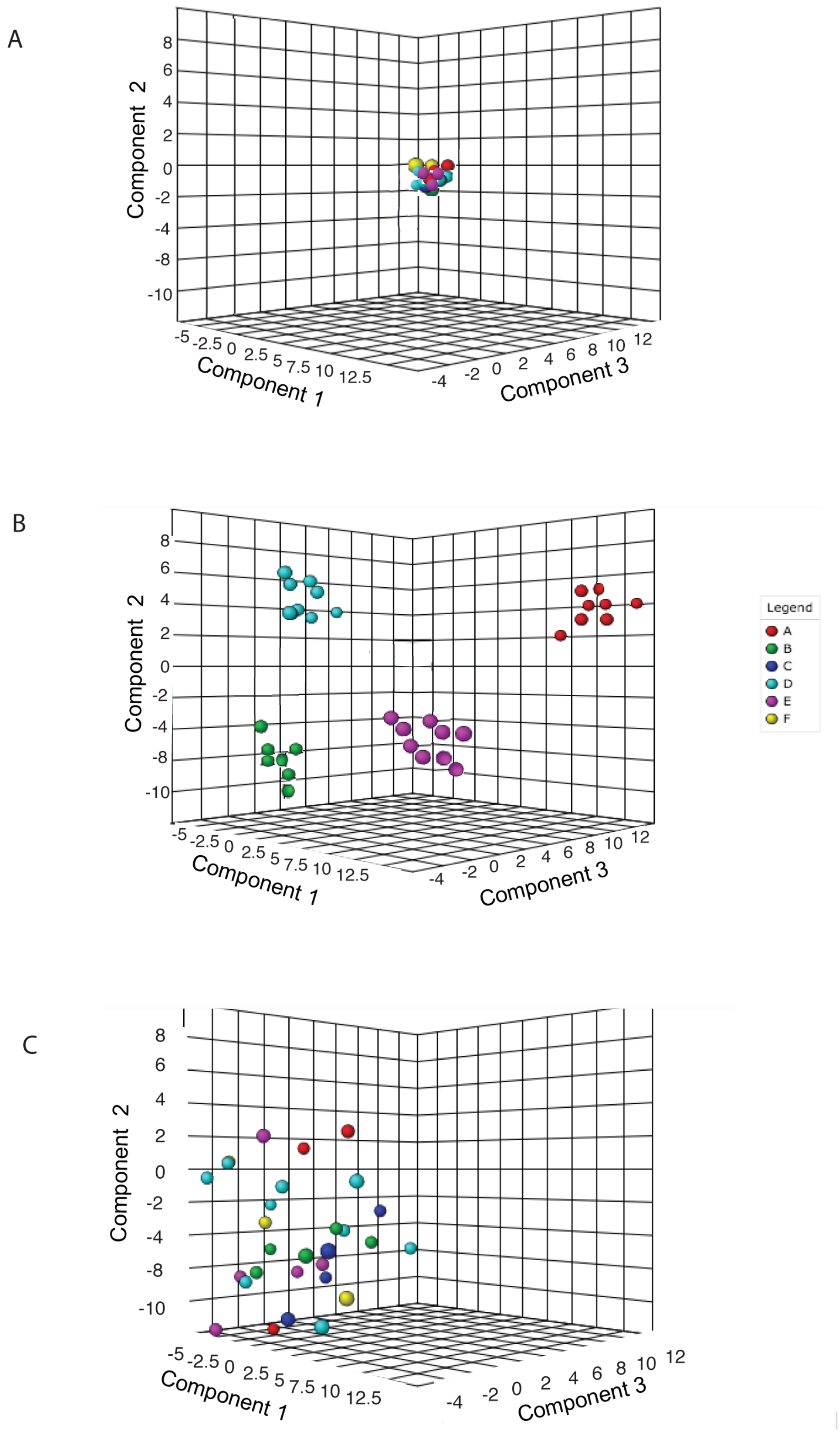

Supplementary Figure 5. Possible hypotheses for chemical variation at sites. (a) same chemistry for all individuals at all sites (b) individuals at each site have the same chemistry, but this differs from the other sites (c) random chemistry within/among sites

A

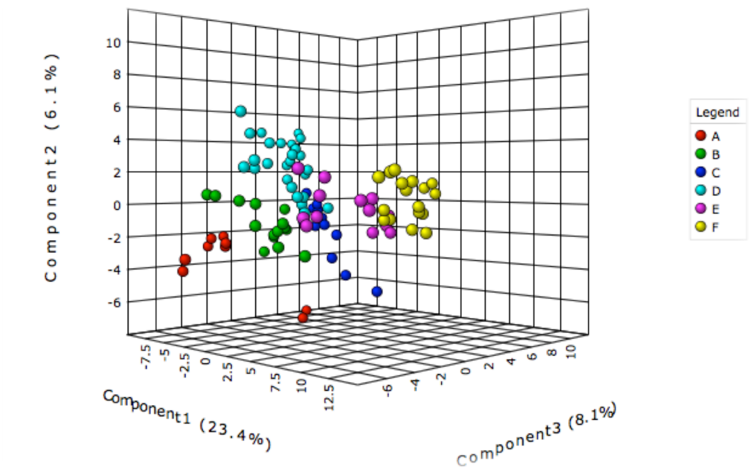

B

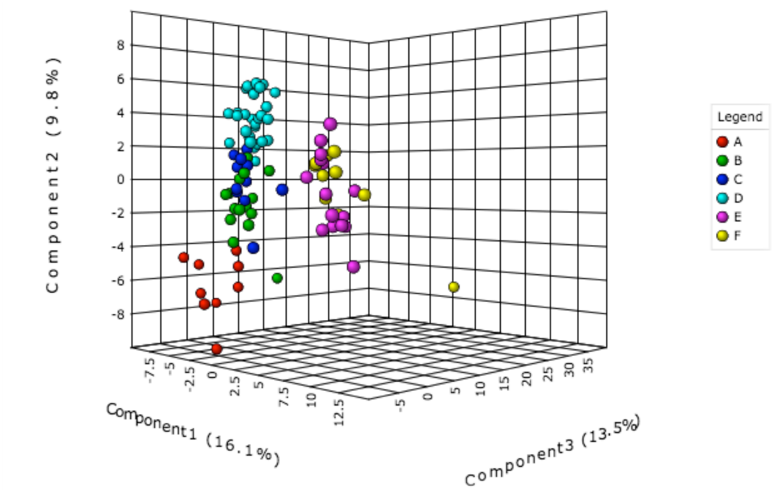

Supplementary Figure 6. PLS-DA of all biological replicates for both extractions. 3D scores plot between selected PCs. The explained variances are shown in brackets. (a) represents all biological replicates of the alkaloid extraction (b) represents all biological replicates of the phenolic extraction

A

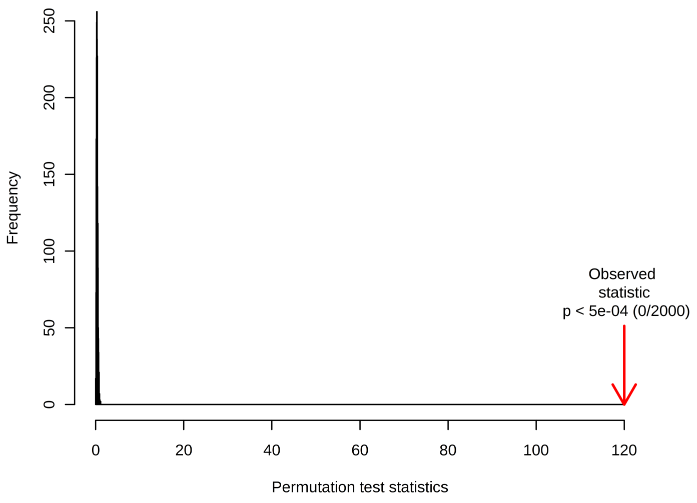

B

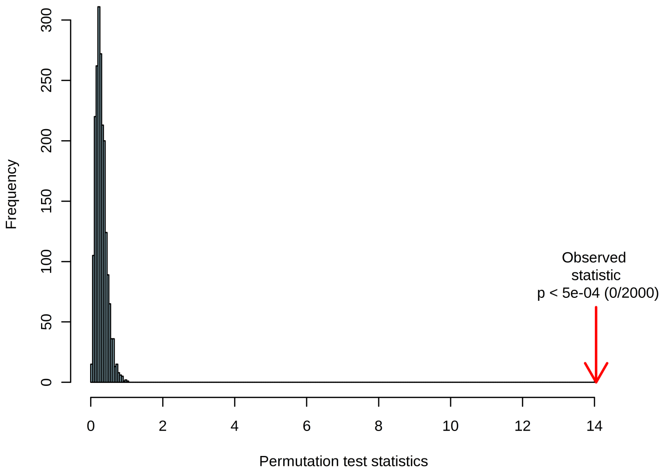

Supplementary Figure 7. PLS-DA model validation by permutation test based on separation distance (B/W) displayed as a histogram. Separation distance is based on the ratio of between-group sum of squares and within-group sum of squares. P-value based on permutation test is p<5e^-04 (0/2000). (a) alkaloid extraction (b) phenolic extraction

Supplementary Table 1. Herbarium sheet references and silica records for individuals used in the study

Genus	Species	Site #	Location	Daniella Allevato Silica Collection Number	Herbarium	Herbarium Sheet Collection Number
Pilocarpus	*pennatifolius*	A	Foz do Iguaçu	5	SP	Coelho, R.L.G.; Allevato, D. 642
Pilocarpus	*pennatifolius*	A	Foz do Iguaçu	6	SP	Coelho, R.L.G.; Allevato, D. 643
Pilocarpus	*pennatifolius*	A	Foz do Iguaçu	7	SP	Coelho, R.L.G.; Allevato, D. 644
Pilocarpus	*pennatifolius*	B	Campo Mourão	75	BH, SPFR	Allevato 75
Pilocarpus	*pennatifolius*	B	Campo Mourão	76	BH, SPFR	Allevato 76
Pilocarpus	*pennatifolius*	B	Campo Mourão	77	BH, SPFR	Allevato 77
Pilocarpus	*pennatifolius*	B	Campo Mourão	78	BH, SPFR	Allevato 78
Pilocarpus	*pennatifolius*	B	Campo Mourão	79	BH, SPFR	Allevato 79
Pilocarpus	*pennatifolius*	C	Campo Mourão B	80	BH, SPFR	Allevato 80
Pilocarpus	*pennatifolius*	C	Campo Mourão B	81	BH, SPFR	Allevato 81
Pilocarpus	*pennatifolius*	C	Campo Mourão B	82	BH, SPFR	Allevato 82
Pilocarpus	*pennatifolius*	C	Campo Mourão B	83	BH, SPFR	Allevato 83
Pilocarpus	*pennatifolius*	D	Parque Municipal Lago Azul	95	BH, SPFR	Allevato 95
Pilocarpus	*pennatifolius*	D	Parque Municipal Lago Azul	96	BH, SPFR	Allevato 96
Pilocarpus	*pennatifolius*	D	Parque Municipal Lago Azul	97	BH, SPFR	Allevato 97
Pilocarpus	*pennatifolius*	D	Parque Municipal Lago Azul	98	BH, SPFR	Allevato 98
Pilocarpus	*pennatifolius*	D	Parque Municipal Lago Azul	99	BH, SPFR	Allevato 99
Pilocarpus	*pennatifolius*	D	Parque Municipal Lago Azul	100	BH, SPFR	Allevato 100
Pilocarpus	*pennatifolius*	D	Parque Municipal Lago Azul	101	BH, SPFR	Allevato 101
Pilocarpus	*pennatifolius*	D	Parque Municipal Lago Azul	102	BH, SPFR	Allevato 102
Pilocarpus	*pennatifolius*	D	Parque Municipal Lago Azul	103	BH, SPFR	Allevato 103
Pilocarpus	*pennatifolius*	E	Cruz do Pedro	110	SPFR	Groppo 2300-A
Pilocarpus	*pennatifolius*	E	Cruz do Pedro	111	SPFR	Groppo 2300-B
Pilocarpus	*pennatifolius*	E	Cruz do Pedro	112	SPFR	Groppo 2300-C
Pilocarpus	*pennatifolius*	E	Cruz do Pedro	113	SPFR	Groppo 2300-D
Pilocarpus	*pennatifolius*	E	Cruz do Pedro	114	SPFR	Groppo 2300-E
Pilocarpus	*pennatifolius*	F	Estacão Ecologica St. Theresa	115	SPFR	Groppo 2302-A
Pilocarpus	*pennatifolius*	F	Estacão Ecologica St. Theresa	116	SPFR	Groppo 2302-B
Pilocarpus	*pennatifolius*	F	Estacão Ecologica St. Theresa	117	SPFR	Groppo 2302-C
Pilocarpus	*pennatifolius*	F	Estacão Ecologica St. Theresa	118	SPFR	Groppo 2302-D
Pilocarpus	*pennatifolius*	F	Estacão Ecologica St. Theresa	119	SPFR	Groppo 2302-E

Supplementary Table 2. Soil analysis. Average of two soil samples for each site analyzed by Instituto Agronômico in Campinas, SP, Brazil

	M.O.	pH	P	K	Ca	Mg	Na	Al	H + Al	S.B.	C.E.C.	V%	S	B	Cu	Fe	Mn	Zn	EC	N
**A**	74	6.4	37	5.9	201	27	0.6	0	16	234.5	250.5	94	0	0.52	4.9	18	42.6	2.8	1.3	3.6
**B**	89	4.35	100.5	2.75	83.5	6.5	0.3	3	139	93.05	232.05	39.5	18.5	0.605	1.95	150.5	22.85	13.55	1.1	16.4
**C**	98	5.8	66	3.05	362	38.5	0.3	1	30	403.85	433.85	92.5	29	0.815	2.45	72.5	29	7.8	0.9	16.75
**D**	96	5.8	57	3.4	328	35	0.3	1	29	366.7	395.7	93	31	0.54	1.4	56	43.6	18	1.5	16.7
**E**	60	5.5	32	2.7	73	18	0.2	0	29	93.9	122.9	76	6	0.27	5.5	22	27.4	1.3	0.7	3.3
**F**	72	6.8	186	4.6	246	36	0.2	0	11	286.8	297.8	96	16	0.43	3.4	24	9.5	8.1	2.2	5.5

Supplementary Table 3. Bioclimatic variables extracted from WorldClim and altitude from DIVA GIS

	Alt	Annual Mean Temp	Mean Diurnal Range	Isothermality	Temp Seasonality	Max Temp Warmest Month	Min Temp Coldest Month	Temp Annual Range	Mean Temp Wettest Qtr	Mean Temp Driest Qtr
**A**	172	21.4958	12.6583	55.5190	359.7882	32.5	9.7	22.8	23.7333	17.0667
**B**	554	20.2167	11.2500	59.2105	291.0118	29.1	10.1	19	23.5000	16.6500
**C**	554	20.2167	11.2500	59.2105	291.0118	29.1	10.1	19	23.5000	16.6500
**D**	585	20.0667	11.2333	59.4356	290.2689	28.9	10	18.9	23.3500	16.5167
**E**	635	21.2167	12.2333	67.5875	204.7097	28.6	10.5	18.1	23.1333	18.4333
**F**	611	21.3500	12.3333	67.3953	203.0114	28.7	10.4	18.3	23.2333	18.5833

	Mean Temp Warmest Qtr	Mean Temp Coldest Qtr	Annual Precipitation	Precipitation Wettest Month	Precipitation Driest Month	Precipitation Seasonality	Precipitation Wettest Qtr	Precipitation Driest Qtr	Precipitation Warmest Qtr	Precipitation Coldest Qtr
**A**	25.7000	17.0667	1729	189	97	17.6189	503	331	463	376
**B**	23.5000	16.5000	1536	188	78	27.6495	516	276	516	317
**C**	23.5000	16.5000	1536	188	78	27.6495	516	276	516	317
**D**	23.3500	16.3833	1555	194	79	28.0923	527	279	527	318
**E**	23.1333	18.3333	1453	257	23	75.6499	732	81	732	101
**F**	23.2333	18.4667	1486	265	24	74.3092	742	88	742	108

Supplementary Table 4. Compounds identified in extractions. (a) alkaloids (b) coumarins

A

Reference ID	LCMS ID	m/z	RT (min)	Molecular Formula	Compound Name
A1	M193T81	193	1.36	C10H12N2O2	13-nor-7(11) dehydropilocarpine
A2	M179T84	179	1.41	C9H10N2O2	4-(3H-imidazol-4-ylmethyl)-3-methyl-5H-furan-2-one
A3	M195T160	195	2.67	C10H14N2O2	Pilocarpidine / isopilocarpidine
A4	M209T167	209	2.79	C11H16N2O2	Pilocarpine / isopilocarpine
A5	M273T193	273	3.21	C15H16N2O3	3-(Hydroxy-phenyl-methyl)-4-(3H-imidazol-4-ylmethyl)-dihydro-furan-2-one
A6	M273T266	273	4.43	C15H16N2O3	3-(Hydroxy-phenyl-methyl)-4-(3H-imidazol-4-ylmethyl)-dihydro-furan-2-one
A7	M259T369	259	6.15	C15H18N2O2	3-Hydroxymethyl-4-(3-methyl-3H-imidazol-4-yl)-1-phenylbutan-1-one
A8	M255T396	255	6.60	C15H14N2O2	3- Benzylidene-4-(3H-imidazol-4-ylmethyl)-dihydro-furan-2-one
A9	M257T407	257	6.79	C15H16N2O2	3-Benzyl-4-(3H-imidazol-4-ylmethyl)-dihydro-furan-2-one
A10	M257T409	257	6.81	C15H16N2O2	3-Benzyl-4-(3H-imidazol-4-ylmethyl)-dihydro-furan-2-one
A11	M255T451	255	7.52	C15H14N2O2	3- Benzylidene-4-(3H-imidazol-4-ylmethyl)-dihydro-furan-2-one

B

Reference ID	LCMS ID	m/z	RT (min)	Molecular Formula	Compound Name
C1	M193T45	193	0.75	C10H8O4	Scopoletin
C2	M187T96	187	1.6	C11H6O3	Psoralen
C3	M147T96	147	1.6	C9H6O2	Coumarin
C4	M217T369	217	6.15	C12H8O4	Xanthotoxin
C5	M247T382	247	6.36	C13H10O5	Isopimpinellin
C6	M245T382	245	6.36	C15H16O3	Osthol
C7	M271T433	271	7.22	C16H14O4	Imperatorin
C8	M203T434	203	7.23	C11H6O4	Imperatorin [271-C5H8]

*LCMS ID refers to fragment/compound IDs in the Raw LCMS Data Table (Supplemental Table 5). Reference ID numbers the identified compounds (A=Alkaloid, C=Coumarin), utilized in Figure 2

Supplementary Table 5. All fragments used in metabolomic analyses. Normalized by dry weight and average of three biological replicates (a) alkaloid extractions (b) phenolic extractions

**See attached excel file*

Supplementary Table 6. Abundance optima of identified compounds associated with environmental variables based on CCA. (a) alkaloids (b) coumarins

A

Compound	Ref ID	P	Mg	V%	MTWQ
13-nor-7(11) dehydropilocarpine	A1	L	L	L	H
4-(3H-imidazol-4-ylmethyl)-3-methyl-5H-furan-2-one	A2	M-H	H	H	L
Pilocarpidine / isopilocarpidine	A3	M-H	H	H	L
Pilocarpine / isopilocarpine	A4	M-H	H	H	L
3-(Hydroxy-phenyl-methyl)-4-(3H-imidazol-4-ylmethyl)-dihydro-furan-2-one	A5	H	M	M-H	H
3-(Hydroxy-phenyl-methyl)-4-(3H-imidazol-4-ylmethyl)-dihydro-furan-2-one	A6	L	L	L	M-L
3-Hydroxymethyl-4-(3-methyl-3H-imidazol-4-yl)-1-phenylbutan-1-one	A7	H	H	H	M-H
3- Benzylidene-4-(3H-imidazol-4-ylmethyl)-dihydro-furan-2-one	A8	L	M-L	M-L	L
3-Benzyl-4-(3H-imidazol-4-ylmethyl)-dihydro-furan-2-one	A9	L	H	H	L
3-Benzyl-4-(3H-imidazol-4-ylmethyl)-dihydro-furan-2-one	A10	L	H	H	L
3- Benzylidene-4-(3H-imidazol-4-ylmethyl)-dihydro-furan-2-one	A11	L	M-L	M-L	M-L

B

Compound	Ref ID	Ca	EC	TS	MTWQ
Scopoletin	C1	H	L	H	H
Psoralen	C2	L	H	L	L
Coumarin	C3	L	L	H	H
Xanthotoxin	C4	M-L	M-L	M-H	M-H
Isopimpinellin	C5	M-L	M-L	M-H	M-H
Osthol	C6	H	L	H	H
Imperatorin	C7	L	H	L	L
Imperatorin [271-C5H8]	C8	H	L	H	H

V% (base saturation), EC (soil electrical conductivity), TS= Temp Seasonality; MTWQ= Mean Temp Wettest Quarter. H=High, L= Low, M=Mid, M-H= Mid-to-High, M-L=Mid-to-Low

Supplementary Table 7. Performance measures for prediction accuracies using cross-validation of PLS-DA using different numbers of components

A. Alkaloid

Measure	1 comps	2 comps	3 comps
**Accuracy**	0.48387	0.45161	0.54839
**R2**	0.6495	0.88856	0.94244
**Q2**	0.50384	0.60991	0.70436

B. Phenolic

Measure	1 comps	2 comps	3 comps
**Accuracy**	0.46667	0.46667	0.60000
**R2**	0.59686	0.82203	0.91282
**Q2**	0.33854	0.43717	0.4544
